# Prevalence and determinants of wasting among under‐5 Egyptian children: Application of quantile regression

**DOI:** 10.1002/fsn3.3144

**Published:** 2022-11-15

**Authors:** Faruq Abdulla, M. M. Abd El‐Raouf, Azizur Rahman, Ramy Aldallal, Mohamed S. Mohamed, Md. Moyazzem Hossain

**Affiliations:** ^1^ Department of Applied Health and Nutrition RTM Al‐Kabir Technical University Sylhet Bangladesh; ^2^ Basic and Applied Science Institute, Arab Academy for Science, Technology and Maritime Transport (AASTMT) Alexandria Egypt; ^3^ School of Computing, Mathematics and Engineering Charles Sturt University Wagga Wagga New South Wales Australia; ^4^ Department of Accounting, College of Business Administration in Hawtat Bani Tamim Prince Sattam bin Abdulaziz University Al‐Kharj Saudi Arabia; ^5^ Department of Mathematics, College of Science Taif University Taif Saudi Arabia; ^6^ Department of Statistics Jahangirnagar University Dhaka Bangladesh; ^7^ School of Mathematics, Statistics and Physics Newcastle University Newcastle upon Tyne UK

**Keywords:** child malnutrition, chi‐square, Egypt, quantile regression, undernutrition, wasting

## Abstract

Wasting is one of the symptoms of malnutrition that has been connected to the deaths of malnourished children. This study was intended to explain the effect of socio‐demographic and economic factors on under‐5 wasting by evaluating their conditional effect across the distribution of weight‐for‐height Z (WHZ) scores using the quantile regression (QR) model. The weighted sample which included 13,680 children under 5 years was taken from the countrywide Egyptian DHS 2014 survey. The results depicted that about 2% of Egyptian children were severely wasted, with the prevalence of wasting being around 8%. It was discovered that across the WHZ distribution, the child's features, maternal characteristics, father's education, and social factors had significant but varied contributions in explaining the wasting status of under‐5 children. It was revealed that female children had a significant weight advantage, notably 0.21 standard deviation (SD) higher weight at the 95th quantile over their male counterparts. The WHZ score was also found to be significantly positively associated with both age and household's wealth status at the lower and upper tails of the WHZ distribution, respectively. Moreover, in comparison with children whose mothers were underweight, those whose mothers were normal or overweight had higher WHZ scores, with a 1.45 SD increase in WHZ scores at the 95th quantile for mothers who were normal weights. Furthermore, the children who were breastfed, whose mothers received antenatal care (ANC) services, and/or who had educated parents had an advantage in terms of WHZ scores than their counterparts. In addition, the children with higher birth order and/or who resided in urban areas had weight disadvantages compared to their counterparts. Therefore, in order to improve children's nutritional status and achieve the Sustainable Development Goals (SDGs) by 2030, the government and public–private owner organizations must work together at the community level focusing on vulnerable groups.

## Graphical abstract

In the target variable, the presence of outliers is confirmed and the authors used the quantile regression (QR) model because it provides the robust estimates than the linear regression model in the presence of outliers. Among children aged under 5 years of age, approximately 8% were wasted and about 2% of children were severely wasted. The highest prevalence of wasting is observed in Frontier governorates (11.19%) and the lowest in Rural Upper Egypt (6.85%). Results of Quantile regression show that at different tails of the weight‐for‐height Z‐scores (WHZ) distribution, a child's WHZ has diverse responses to several socioeconomic and demographic variables. To enrich the nutritional status of children along with achieving Sustainable Development Goals (SDGs) by 2030, a collaborative approach should necessarily be taken by the government and public–private owner organizations at the community level in Egypt.

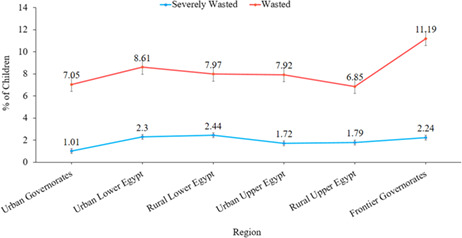

## 1 INTRODUCTION

Undernourished children have weakened immunity and impaired cognitive function, which leads to their poor health outcomes, loss of future productivity, and low academic performance (Anato, 2022; WHO & UNICEF, 2009). Consequently, undernutrition is responsible for almost half (45%) of all deaths in under‐5 children worldwide (Anato, 2022; FAO et al., 2017). Wasting or acute malnutrition refers to a child's thinness due to a lack of nutritional intake and/or illness (Karlsson et al., 2022; Motbainor & Taye, 2019). It is one of the most severe manifestations of the global public health concern termed as undernutrition in the developing countries (Motbainor & Taye, 2019). There are many gravely detrimental impacts of wasting on children's health, with the highest risk of mortality (Black et al., 2013; Ghosh‐Jerath et al., 2017; Harding et al., 2018; Tickell et al., 2017). Severe wasting is one of the leading hazards to child survival, resulting in one in five deaths of under‐5 children worldwide, killing more than 1 million children every year (UNICEF, 2022). Annually, 8 million deaths are anticipated to be caused by wasting, with severe wasting responsible for 60% of these deaths in low‐ and middle‐income countries (LMICs) (Anato, 2022; Black et al., 2013).

There were 45.4 million wasted children worldwide under the age of 5 in 2020, with 13.6 million of those being severely wasted (Anato, 2022; UNICEF et al., 2020). Africa has the second highest prevalence of wasting (27%) in the world (Anato, 2022). In Egypt, the prevalence of wasting in children under the age of 5 is 9.5% which is more than 1.5 times the average rate of wasting (6.0%) for the African region (Global Nutrition Report, 2022). In addition, the socioeconomic effects of COVID‐19 might also contribute to an increase in the prevalence of wasting (Headey et al., 2020). One of the “United Nation's (UN) Sustainable Development Goals (SDGs)” to be achieved by 2030 is “Zero Hunger” with the target of “end all forms of malnutrition by 2030” (United Nations, 2015). However, Egypt is indeed far from reaching its goal of reducing childhood wasting (Global Nutrition Report, 2022). Consequently, the existing policies and intervention programs against wasting should be redesigned focusing on the really vulnerable groups in order to lessen the burden of wasting in Egypt.

The socio‐demographic and economic profiles of all the wasted children and their families are not the same. Therefore, earlier studies explored that many child's features—including sex, age, birth order, low birth weight, inadequate breast feeding, infectious diseases, vaccination, insufficient energy and micronutrient intake, and inappropriate complementary feeding practices; parental characteristics—such as education, low nutritional knowledge and awareness, mother's age and body mass index (BMI), ANC visits during pregnancy period, and birth spacing; as well as some social factors—including types of living area, wealth status, and food availability; environmental factors and many more have significant contribution in explaining the wasting status of children under the age of 5 (Ali et al., 2017; Anato, 2022; Biadgilign et al., 2016; Black et al., 2013; Harding et al., 2018; Karlsson et al., 2022; Khan et al., 2019; Li et al., 2020; Manda et al., 2015; Molla et al., 2022; Motbainor & Taye, 2019; Mukabutera et al., 2016; Ntenda & Chuang, 2018; Rahman et al., 2008; Rahman & Chowdhury, 2007; Tigga & Sen, 2016; Vollmer et al., 2017).

However, few studies have been conducted on wasting in Egypt. Abdel Wahed et al. (2017) conducted a cross‐sectional study based on school‐based survey data and identified the significant determinants of malnutrition through stepwise logistic regression analysis in Fayoum Governorate, Egypt (Abdel Wahed et al., 2017). In a separate study by Khatab (2010), the author discussed the associated factors of malnutrition by employing geoadditive Gaussian and latent variable models in Egypt (Khatab, 2010). Using both simple and multiple logistic regression models, El‐Sayed et al. (2001) determined the contributing factors to malnutrition among preschool children in Alexandria, Egypt (El‐Sayed et al., 2001).

Nevertheless, the quantile regression (QR) approach can be utilized to describe the diverse association through the distinct percentiles of the conditional distribution of the WHZ score (Sharaf et al., 2019), and the QR outcomes might be helpful in designing appropriate policy and intervention measures, especially in the presence of outliers and nonnormality in the dataset (Olsen et al., 2012; Yeh et al., 2009). Several researchers applied the QR model to examine the core socio‐demographic factors of child nutritional status but all of those studies have been conducted in the out context of Egypt (Aturupane et al., 2011; Borooah, 2005; Fenske et al., 2013; S Hossain et al., 2022; Rahman & Hossain, 2022; Sharaf et al., 2019).

The focus of this study was to use the QR approach to quantify the heterogeneous effects of socio‐demographic and economic factors across the conditional distribution of WHZ scores of children under the age of 5 in the context of Egypt. The “weight‐for‐height” Z‐score was employed to measure the child's wasting status. All of the analyses were done based on the Egyptian Demographic and Health Survey 2014 (EDHS‐2014). The findings of this research would help to develop need‐based policy and intervention strategies for reducing the burden of wasting in Egypt.

## 2 METHODS

### 2.1 Data collection

This research was based on the results of the Egyptian DHS survey, which was conducted in 2014. The EDHS‐2014 study was a nationwide household survey that provided a wide range of demographic, health, and nutrition data. The sampling frame was created on the 2006 Egypt Population Census which was updated by the “Central Agency for Public Mobilization and Statistics (CAPMAS)”. For the EDHS‐2014, the sample was chosen in four steps. The Primary stage units (PSUs) were chosen from 926 shiakhas/villages in the first stage. In the second stage, each PSU was divided into parts (each part included about 1000 households) and each part was subdivided further into segments (each segment included about 200 households and was called a cluster). Two to three clusters were chosen in the third stage, with a probability proportional to the corresponding PSUs. North and South Sinai governorates were not covered because of security concerns; as a result, the total number of PSUs is 884. A “systematic random sampling process” was employed to select 15 households from each cluster, with a total of 33 households per PSU. Children under the age of 5 were included in this study. The authors used a weighted sample in the final analysis. The details of sampling procedure is available in the published EDHS‐2014 report [Egypt] El‐Zanaty and Associates [Egypt] and ICF International, (Ministry of Health and Population, 2015).

### 2.2 Variables

A child was termed wasted if his/her weight‐to‐height (WHZ) ratio was higher than 2 SD (standard deviation) below the reference population's median weight‐to‐height ratio. The weight‐to‐height Z (WHZ)‐score‐based anthropometric indicator was used as the target variable, and several child characteristics such as sex (male, female), age (≤6, 7–12, 13–23, 24–35, 36–47, and 48–59 months), duration of breastfeeding (never breastfed, ≤12, 13, or more months), birth order (first, second, third, fourth, or higher); maternal attributes such as age (≤18, 19–24, 25–34 , ≥35 years), education (no education, primary, secondary, or higher), and BMI (underweight (<18.5), normal (18.5–24.9), overweight (≥25)); father's education (no education, primary, secondary, or higher), and attributes related to household, community along with health were the explanatory variables in this study. All categories of the covariates are presented in Table 1. The availability of the EDHS dataset, self‐efficacy, as well as related existing research, led to the selection of the variables employed in this investigation (Ahsan et al., 2017; Akram et al., 2018; Aturupane et al., 2011; Biadgilign et al., 2016; Borooah, 2005; Chowdhury et al., 2018; Das & Gulshan, 2017; Fenske et al., 2013; S Hossain et al., 2022; Manda et al., 2015; Mohsena et al., 2015; Mukabutera et al., 2016; Ntenda & Chuang, 2018; Rabbani et al., 2016; Rahman et al., 2008; Rahman & Chowdhury, 2007; Rahman & Hossain, 2022; Sharaf et al., 2019; Vollmer et al., 2017).

**TABLE 1:** Percent distribution of child wasting by background characteristics, EDHS‐2014, *n* = 13,680

Background characteristics	Percent	Weight‐for‐height (wasted) in %		*p*‐Value of chi‐square
		Z‐score <−3 SD	Z‐score <−2 SD	
Child's sex				
Male	52.28	2.03	7.77	.054
Female	47.72	1.98	7.44	
Age of the child (Months)				
≤6	10.51	3.20	10.08	<.001
7–12	11.14	1.90	10.03	
13–23	19.00	2.58	10.12	
24–35	20.84	2.14	7.33	
36–47	20.81	1.47	5.76	
48–59	17.69	1.16	4.46	
Birth order				
1st	30.46	1.97	7.18	.075
2nd–3rd	50.12	1.87	7.64	
4th or higher	19.42	2.37	8.21	
Duration of breastfeeding (Months)				
Never breastfed	5.01	2.04	7.29	<.001
≤12	19.29	2.54	10.00	
13 or more	75.70	1.85	7.02	
Mother's age (Years)				
≤18	1.18	1.85	6.79	.037
19–24	23.26	2.20	8.27	
25–34	60.05	2.02	7.61	
≥35	15.51	1.65	6.69	
Mother's BMI				
Underweight (<18.5)	0.32	0.00	4.65	.033
Normal (18.5–24.9)	20.51	2.25	8.91	
Overweight (≥25)	79.18	1.94	7.29	
Mother's education level				
No education	17.68	1.90	7.73	.001
Primary	8.63	1.86	5.68	
Secondary or higher	73.69	2.03	7.81	
Father's education level				
No education	12.56	2.04	8.79	.047
Primary	13.48	1.95	6.12	
Secondary or higher	73.96	2.00	7.68	
Type of place of residence				
Rural	69.31	2.16	7.53	.020
Urban	30.69	1.64	7.84	
Place of delivery				
With health facility	86.69	2.00	7.70	.472
Respondent's home	13.31	1.90	6.90	
Number of ANC visits				
None	18.80	1.52	6.42	<.001
1–3	7.45	1.96	6.28	
4–7	24.56	1.70	7.32	
8 or more	49.20	2.33	8.42	
Had diarrhea recently				
No	85.99	2.03	7.68	.863
Yes	14.01	1.77	7.25	
Had fever in last 2 weeks				
No	73.82	2.10	7.80	.321
Yes	26.18	1.60	7.00	
Had cough in last 2 weeks				
No	72.38	2.20	7.90	.238
Yes	27.62	1.50	7.00	
Received BCG				
No	1.38	1.60	7.45	.986
Yes	98.62	2.00	7.61	
Wealth index				
Poorest	17.91	1.60	6.60	<.001
Poorer	19.58	1.80	7.20	
Middle	24.97	2.30	8.40	
Richer	21.01	2.80	8.90	
Richest	16.53	1.20	6.40	
Total		2.00	7.61	

### 2.3 Statistical methods

To determine the findings of this study, descriptive statistics, percent distribution, and the Chi‐square were used. The boxplot was employed to detect the presence of outliers and the estimates of mean and variance were influenced by outliers (Hossain, 2016, 2017); however, in this circumstance, QR provided the robust results. Finally, the quantile regression (QR) model was used for modeling purpose that was initially introduced by Koenker & Bassett, 1978, and nowadays, it is extensively applied in various research areas, particularly in Statistics, Econometrics, and public health (Aturupane et al., 2011; Borooah, 2005; Fenske et al., 2013; Hossain et al., 2021; Hossain & Majumder, 2019; Koenker & Bassett, 1978; Rahman & Hossain, 2022; Sharaf et al., 2019). Suppose, Y be a random variable having cumulative distribution function *F*_y_(*y*) i.e., $F_{Y}\left(y\right)=P\left(Y\leq y\right)$. Then, the quantile function can be presented in the following way $Q_{Y}\left(\tau \right)=F_{Y}^{-1}\left(\tau \right)=\inf\left\{y:F_{Y}\left(y\right)\geq \tau \right\},\tau \in \left[0,1\right]$.

The parametric form of the quantile regression model can be stated as $y_{i}=\beta_{0\tau }+\beta_{1\tau }x_{1i}+\ldots +\beta_{\mathrm{k\tau}}x_{\mathrm{ki}}+\varepsilon_{\mathrm{i\tau}}\forall i\in \left\{1,2,\ldots ,n\right\}$, where, $\beta_{0\tau },\beta_{1\tau },\ldots ,\beta_{\mathrm{k\tau}}$ are the coefficients that may differ on τ. The coefficients for different quantiles of the model are calculated by solving the following equation,

$$\begin{array}{l} \arg\min\left\{\sum_{y_{i>A}}\tau \left|y_{i}-\beta_{0\tau }-\beta_{1\tau }x_{1i}-\ldots -\beta_{\mathrm{k\tau}}x_{\mathrm{ki}}\right|+\sum_{y_{i<A}}\left(1-\tau \right)\left|y_{i}-\beta_{0\tau }-\beta_{1\tau }x_{1i}-\ldots -\beta_{\mathrm{k\tau}}x_{\mathrm{ki}}\right|\right\}\\ \left(\beta_{0\tau },\ldots ,\beta_{\mathrm{k\tau}}\right)\in R^{2} \end{array}.$$

The authors considered 0.10, 0.25, 0.50, 0.75, and 0.95 quantile levels based on the previous studies (Hossain et al., 2021; Hossain et al., 2022; Hossain & Majumder, 2019; Rahman & Hossain, 2022; Sharaf et al., 2019).

## 3 RESULTS

**FIGURE 1:**
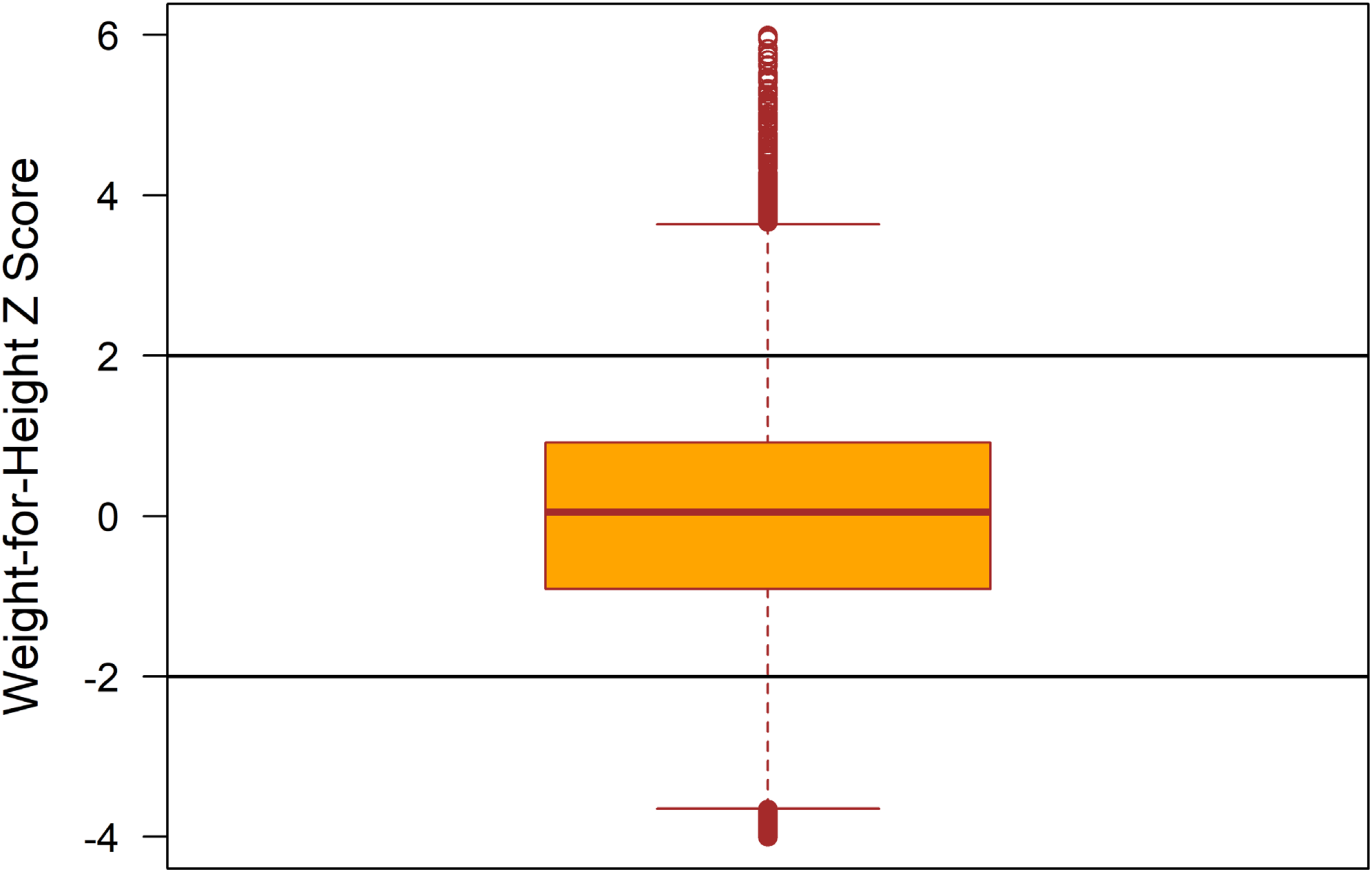
Boxplot of the weight‐for‐height Z‐scores (WHZ) of under‐5 children

The boxplot presented in Figure 1 depicted that there were outliers in the WHZ scores of the under‐5 children in Egypt. This was the motivation working behind using QR regression analysis in this study.

Table 1 illustrates the prevalence of wasting among the children under 5 years of age according to selected socio‐demographic and economic characteristics. The findings revealed that the prevalence of wasting (WHZ‐score < −2 SD) among children aged under 5 years was approximately 8% and only about 2% of children were severely wasted (WHZ‐score < −3 SD) in Egypt. The results also showed that all characteristics analyzed have a significant association with the wasting outcome, with the exception of the place of delivery, child's health status (had diarrhea, fever, or cough in the past 2 weeks), and receiving status of BCG vaccination. The prevalence of wasting was varied by the age of the child and it was seen that the percent of wasted children decreased as their age increased. The prevalence of wasting was almost the same among children aged under 2 years; however, after the age of 2 years, the prevalence of wasted children gradually declined. Moreover, the highest (3.2%) prevalence of severely wasted children was observed among children aged 6 months or younger and the lowest (1.16%) rate of severely wasted children was reported for ages 4–5 years. A higher prevalence of wasting was also observed among the children who had higher birth order. The prevalence of severely wasted children was slightly higher among rural children than their counterparts. Moreover, the parental education had a strong association with childhood wasting. The wealth quintile was also associated with wasting and the lowest prevalence of wasting was found among the children who came from the richest families (Table 1).

**FIGURE 2:**
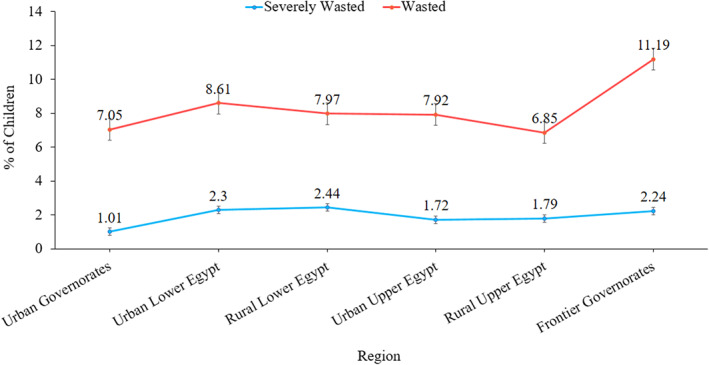
Prevalence of wasting among children region in Egypt

The rates of wasting varied by region, with ‘Rural Upper Egypt’ and ‘Frontier Governorates’ having the lowest (6.85) and highest (11.19) rates of wasting, respectively. For all regions, the proportion of severely wasted children was around 2%. The overall scenario was slightly better in ‘Urban Governorates’ and ‘Rural Upper Egypt’ compared to other regions (Figure 2).

In the bivariate analysis, the identified significant determinants were incorporated in the QR analysis. It was observed that the Pseudo *R* ^2^ increased from lower to upper quantile levels, indicating that the effect of the related factors on WHZ score increased from lower to upper quantiles. Notably, the considered factors were found to be less important to explain the variation in the WHZ score. It was discovered that the QR estimates of the sex of the child at all chosen percentile levels (10%, 25%, 50%, 75%, and 95%) were found to be significant. Furthermore, in comparison with the child's age group of less than or equal to 6 months, all other age groups' coefficients were negative and statistically significant at the majority of the percentiles, indicating a negative influence on the WHZ score. Additionally, children with higher birth order had larger WHZ score than those who were the first children of their parents. Duration of breastfeeding and mother's age were also found to have no significant effect on the WHZ score. Moreover, the QR estimates demonstrated a positive association between the mother's BMI and WHZ, even though the coefficients had a negative sign at the lowest percentile but switched sign at higher percentiles with strong significance and higher magnitude (Table 2).

**TABLE 2:** Results of QR analysis of child wasting and selected covariates (dependent variable: WHZ), EDHS‐2014

Characteristics	Labels	Quantile regression estimate				
		Q10	Q25	Q50	Q75	Q95
Child's sex	Male (Ref.)					
	Female	0.076* (0.047)	0.112** (0.046)	0.097*** (0.026)	0.108*** (0.03)	0.211*** (0.056)
Child's age (Months)	≤6 (Ref.)					
	7–12	−0.193** (0.091)	−0.426*** (0.096)	−0.464*** (0.063)	−0.497*** (0.065)	−0.517*** (0.134)
	13–23	−0.295* (0.122)	−0.467*** (0.141)	−0.389*** (0.106)	−0.592*** (0.128)	−0.505** (0.191)
	24–35	−0.001 (0.235)	−0.286** (0.135)	−0.297** (0.095)	−0.699*** (0.113)	−0.841*** (0.16)
	36–47	0.167 (0.219)	−0.174 (0.147)	−0.249** (0.099)	−0.687*** (0.129)	−0.915*** (0.151)
	48–59	0.365* (0.123)	−0.095 (0.135)	−0.249** (0.111)	−0.683*** (0.125)	−0.682** (0.203)
Birth order	1st (Ref.)					
	2nd–3rd	−0.093** (0.044)	0.02 (0.054)	0.012 (0.034)	−0.025 (0.043)	−0.087* (0.064)
	4th or higher	−0.116 (0.106)	−0.073 (0.062)	−0.085* (0.05)	−0.201*** (0.056)	−0.201** (0.088)
Duration of breastfeeding (Months)	Never breastfed (Ref.)					
	≤12	0.089 (0.315)	0.017 (0.122)	0.178** (0.086)	−0.046 (0.153)	0.128 (0.162)
	13 or more	0.048 (0.163)	0.034 (0.042)	0.053 (0.051)	−0.009 (0.065)	0.113 (0.17)
Mother's age (Years)	≤18 (Ref.)					
	19–24	0.03 (0.23)	0.034 (0.265)	0.091 (0.133)	−0.094 (0.194)	−0.012 (0.322)
	25–34	0.043 (0.219)	−0.041 (0.278)	0.028 (0.123)	−0.103 (0.192)	−0.177 (0.354)
	≥35	0.046 (0.219)	−0.073 (0.247)	−0.003 (0.138)	−0.076 (0.205)	−0.153 (0.351)
Mother's education level	No education (Ref.)					
	Primary	0.069 (0.11)	0.002 (0.094)	−0.006 (0.077)	0.06 (0.064)	−0.2** (0.082)
	Secondary or higher	−0.058 (0.08)	−0.057 (0.059)	−0.047 (0.04)	−0.028 (0.051)	−0.043 (0.085)
Mother's BMI	Underweight (<18.5) (Ref.)					
	Normal (18.5–24.9)	−0.183 (0.23)	0.223 (0.202)	0.62*** (0.084)	0.929*** (0.222)	1.452*** (0.368)
	Overweight (≥25)	−0.171 (0.227)	0.264* (0.187)	0.676*** (0.084)	1.042*** (0.217)	1.527*** (0.383)
Father's education level	No education (Ref.)					
	Primary	0.308** (0.105)	0.255*** (0.065)	0.168** (0.068)	0.108** (0.044)	0.09 (0.092)
	Secondary or higher	0.295** (0.102)	0.158*** (0.05)	0.088** (0.046)	−0.015 (0.038)	−0.101 (0.092)
Type of place of residence	Rural (Ref.)					
	Urban	−0.081 (0.095)	−0.202*** (0.062)	−0.243*** (0.055)	−0.128** (0.058)	−0.103 (0.128)
Number of ANC visits	None (Ref.)					
	1–3	0.054 (0.094)	0.013 (0.071)	−0.009 (0.054)	−0.028 (0.055)	−0.09 (0.155)
	4–7	0.049 (0.054)	0.015 (0.048)	0.055* (0.035)	0.016 (0.038)	−0.014 (0.099)
	8 or more	−0.23*** (0.065)	−0.116** (0.045)	−0.055 (0.045)	−0.049 (0.046)	0.079 (0.086)
Wealth index	Poorest (Ref.)					
	Poorer	0.019 (0.055)	−0.026 (0.042)	−0.027 (0.032)	−0.077* (0.056)	−0.139* (0.089)
	Middle	−0.259** (0.089)	−0.106* (0.063)	−0.026 (0.037)	−0.007 (0.062)	−0.034 (0.119)
	Richer	−0.42*** (0.103)	−0.108* (0.083)	0.07* (0.053)	0.061 (0.086)	0.053 (0.139)
	Richest	−0.144 (0.152)	0.022 (0.104)	0.174** (0.075)	0.113 (0.114)	0.202* (0.156)
Constant		−1.779*** (0.352)	−0.895* (0.377)	−0.451** (0.158)	0.657** (0.341)	1.683** (0.534)
Pseudo *R* ^2^		0.103	0.288	0.212	0.315	0.329

*Note*: Ref. stands for reference; standard errors are presented in parentheses.

* *p* < .1.

** *p* < .05.

*** *p* < .01.

**FIGURE 3:**
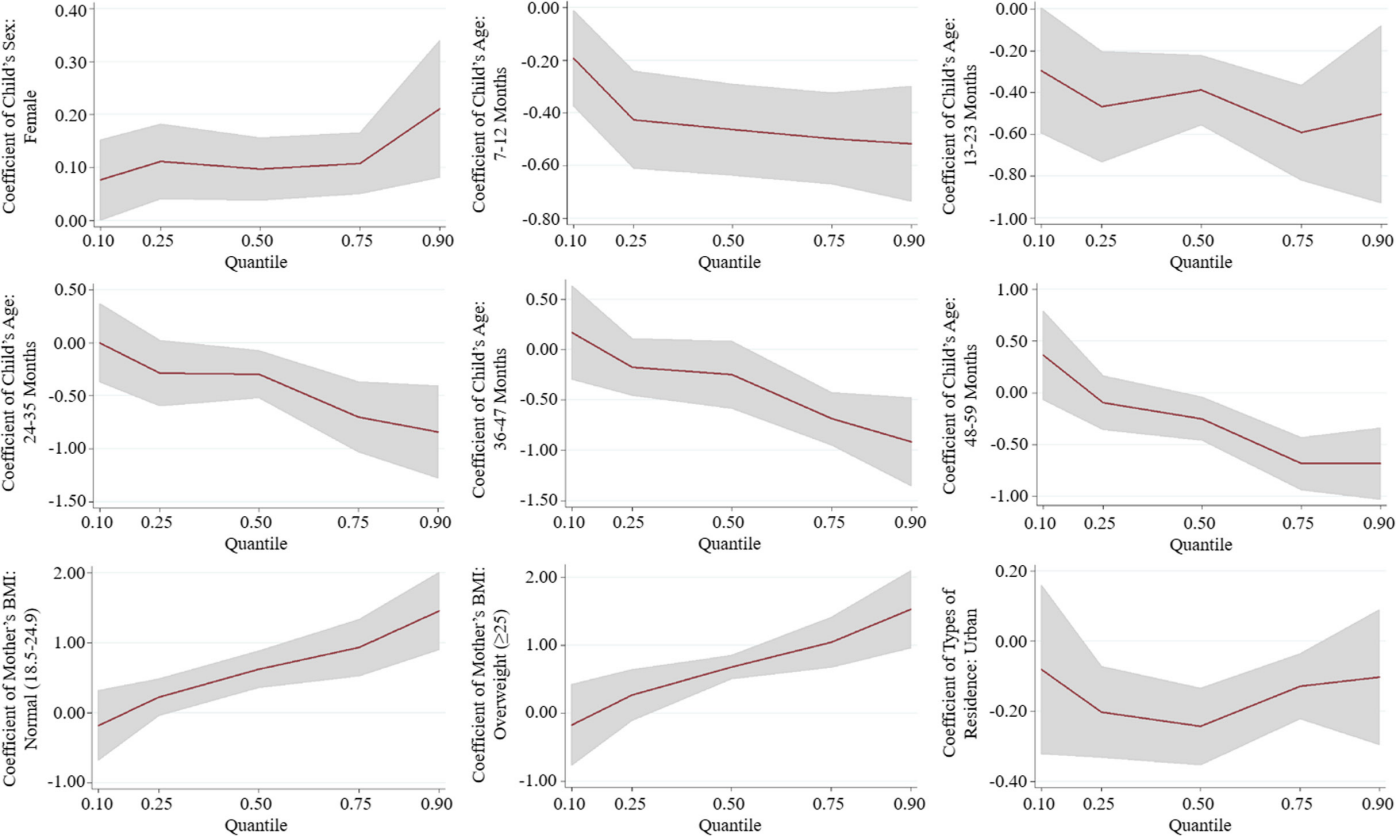
Quantile regression estimates for some selected determinants of WHZ score

The coefficient of the father's educational status decreased in magnitude from lower to upper quantiles of the conditional WHZ distribution, indicating that father's educational status had larger effect on the WHZ score at the lower tail of the WHZ distribution in Egypt. Contrary to what may be expected, children who lived in urban areas were scored worse on the WHZ indicator than those who lived in rural areas. As well, a ridiculous scenario was also demonstrated by the significant coefficients against ANC visits. In addition, the wealth index had a statistically significant positive association with WHZ at some percentiles; interestingly, children who came from the middle, richer, and richest families had a lower WHZ than children who came from the poorest families at the 10th percentile (Table 2). The heterogeneous effects along with 95% confidence interval of some selected determinants throughout the entire conditional WHZ distribution are illustrated in Figure 3.

## 4 DISCUSSION

This study was aimed to assess the conditional heterogeneous effects of different associated determinants on wasting among children aged under 5 years in Egypt considering the EDHS‐2014 dataset. Both bivariate and quantile regression analysis demonstrated that childhood wasting was significantly associated with sex, age, birth order of the children, duration of breastfeeding, maternal characteristics (education and BMI), father's education, place of residence, number of ANC visits during pregnancy, and wealth quintile. These characteristics were also reported as explanatory factors for WHZ scores of children under the age of 5 in several prior studies (Ali et al., 2017; Anato, 2022; Biadgilign et al., 2016; Black et al., 2013; Harding et al., 2018; Karlsson et al., 2022; Khan et al., 2019; Li et al., 2020; Manda et al., 2015; Molla et al., 2022; Motbainor & Taye, 2019; Mukabutera et al., 2016; Ntenda & Chuang, 2018; Rahman et al., 2008; Rahman & Chowdhury, 2007; Tigga & Sen, 2016; Vollmer et al., 2017). On the other hand, place of delivery, mother's age, having recent diarrhea, fever, cough, and the status of receiving BCG vaccination did not show any significant association with childhood wasting.

It was observed that female children had a weight advantage than male counterpart at all considered quantiles. Earlier research made a similar conclusion with 26% higher odds of wasting in male children than their female counterpart and postulated biological and social factors for explaining the sex difference in wasting (Thurstans et al., 2020). If the excessive demand for nutrients due to a greater biologically programmed trajectory in male children is not met adequately, they will experience deficit earlier and much more than female children which may partially describe the link between sex and wasting (WHO Multicentre Growth Reference Study Group & de Onis, 2006). The age of a child was found to be strongly connected to wasting in children below 5 years of age, with the WHZ score decreased as the child's age increased. Our findings are also supported by previous research (Darteh et al., 2017; Islam et al., 2020). This could be due to the fact that children are more vulnerable to infections throughout their first year of life. The children having higher birth order had a weight disadvantage than the first child of the parent. The plausible explanation, for this reason, maybe the parents are probably more careful of their first child.

Parental education had a significant influence on the various points of the conditional WHZ distribution of under‐5 children, this result is consistent with the findings from previous studies (El et al., 2009; Semba et al., 2008; Sharaf et al., 2019; Sharaf & Rashad, 2016). Nutritional status has long been connected to socioeconomic status, which is often defined by income and education level. The amount spent on nutritionally balanced foods is influenced by one's income. Moreover, educational attainment influences nutritional knowledge and awareness regarding health, environment, childcare, food habits, physical activity, and so on (Sharaf et al., 2019). The relationship between a father's education and WHZ score could be explained by the fact that educated fathers have educated spouses, leading to improved care practices for their children. Therefore, nutrition and health‐related education should be integrated into the education system in Egypt.

Except at the 10th quantile, the children of mothers with normal or overweight had an advantage in terms of weight than those of mothers with underweight. In several earlier research, maternal BMI was also identified as the most predictive variable of children's WHZ score (Ali et al., 2017; Khan et al., 2019;Li et al., 2020; Tigga & Sen, 2016). Malnutrition among children may be attributed to the intrauterine intergenerational transmission of low maternal BMI during pregnancy and/or biological consequences of maternal malnutrition during lactation (Li et al., 2020; Tigga & Sen, 2016). The mother–child nutritional relationship may continue from generation to generation. Therefore, it is suggested that nutrition interventional programs in Egypt should take into consideration maternal socio‐demographic features in order to improve the nutritional status of under‐5 children.

In Egypt, the urban children had a weight disadvantage compared to their rural counterpart as well as the duration of breastfeeding and number of ANC visits had no significant effect on the WHZ score. These findings are at contrasts with what is already known (Ip et al., 2007; Lawrence & Lawrence, 2010; Oddy, 2001; Sharaf et al., 2019). The reason behind these fascinating scenarios was not understood in this study; therefore, the authors suggest further study focusing on these issues. A significant positive association between household's wealth index and WHZ score was found only at upper quantiles. Possible reason behind this finding may be the wealthier households can manage to pay for better medical care and additional nutritious food as well as ensure a healthier living environment (Islam et al., 2020; Sharaf et al., 2019). The prevalence of wasting is varied by region. Probably, geographic disparities, such as road and railway networks, products availability and costs, nutritional diversity and customs, conflicts, and land scarcity, could explain these within‐country variances (Rahman & Hossain, 2022; Sharaf et al., 2019; Sharaf & Rashad, 2016).

## 5 STRENGTHS AND LIMITATIONS

The strength of this study was the novelty of the work and considering the country representative EDHS‐2014 data. This was a cross‐sectional study; therefore, the causal inference was not possible. Furthermore, there may be spatial and temporal variation exist in the prevalence of wasting but the authors did not consider it here.

## 6 CONCLUSIONS

This study discovered that sex, age, birth order of the children, duration of breastfeeding, mother's BMI, parental education, place of residence, number of ANC visits during pregnancy, and household's wealth status had varied effect (in magnitude and direction) throughout the conditional distribution of WHZ score of the children under the age of 5 in Egypt. According to the findings of this study, it is recommended to update the current undernutrition remedial policies and strategies as well as develop some need‐based intervention measures focusing on the vulnerable populations in order to lessen the burden of wasting. The authors are confident that the outcomes of this paper would help policymakers in accelerating the achievement of the SDG‐2 in Egypt.

## CONFLICT OF INTEREST

The authors have declared that no competing interests exist.

## ETHICAL APPROVAL

This study was based on an existing public domain survey dataset that is freely available in online after removing all identifier information of the respondents through a registration process. The survey was approved by the Ethics Committee of the ICF Macro at Calverton in the USA and by the Ethics Committee in Egypt.

## Data Availability

The access link of dataset is http://dhsprogram.com/data/available‐datasets.cfm.
